# Supplementary material for: Pre-labor rupture of membranes and associated factors among pregnant women admitted to the maternity ward, Northwest Ethiopia
Source: PLOS Glob Public Health. 2023 Mar 8;3(3):e0001702. doi: 10.1371/journal.pgph.0001702 (PMC10022274; doi:10.1371/journal.pgph.0001702)
Supplement: Attachment — Submitted filename: Response to Reviewers.docx [file pgph.0001702.s002.docx]

**Response to Reviewers point by point**

We are glad for your constructive comments and suggestions. We are also learned a lot from these substantial scientific comments to improve the quality of this manuscript. We are addressed your comments and suggestions as stated below. The manuscript was revised as indicated in the highlights and track changes in the document.

**Journal Requirements:**

1. Please provide the full name of the institutional review board (IRB) who approved your study.

**Thank you!** We are accepted your comment and included in the document page 22 lines 389-390.

2. In the online submission form, you indicated that “The datasets used and/or analyzed during the current study are available from the corresponding author upon reasonable request.” All PLOS journals now require all data underlying the findings described in their manuscript to be freely available to other researchers, either 1. In a public repository, 2. Within the manuscript itself, or 3. Uploaded as supplementary information.
   This policy applies to all data except where public deposition would breach compliance with the protocol approved by your research ethics board. If swz\!your data cannot be made publicly available for ethical or legal reasons (e.g., public availability would compromise patient privacy), please explain your reasons by return email and your exemption request will be escalated to the editor for approval. Your exemption request will be handled independently and will not hold up the peer review process, but will need to be resolved should your manuscript be accepted for publication. One of the Editorial team will then be in touch if there are any issues.

**Thank you!** We are accepted your comment and attached the dataset as supplementary information.

3. Please review your reference list to ensure that it is complete and correct. If you have cited papers that have been retracted, please include the rationale for doing so in the manuscript text, or remove these references and replace them with relevant current references. Any changes to the reference list should be mentioned in the rebuttal letter that accompanies your revised manuscript. If you need to cite a retracted article, indicate the article’s retracted status in the References list and also include a citation and full reference for the retraction notice.

**Thank you!** For your interesting scientific comments and we are corrected as your suggestion. The reference (PARRY S: **Mechanism of disease premature rupture of the fetal membranes** *Kofinas Perinat* 2010, **1**:1-13.) was deleted from the reference lists.

**Additional Editor Comments:**

1. This manuscript is meeting PGPH Journal's publication criteria in the basis and quite interesting to clarify better the problem of PROM and associated factors in obstetric care. Before your manuscript will be sent for peer review, the formula of sample size calculation, in the Sampling procedure section at page 93, should be showed by the formula (not presenting in text) following the cross sectional study design and not used OR for sample estimation. You could use p estimation from previous studies to calculate sample size for your study.

**Thank you!** For your critical citation, the sample size calculation using single population formula was checked at the beginning of the study. For your further information we have stated below.

At the initial sample size determination, we are trying to calculate sample size using single population proportion formula taking the proportion of pregnant mothers with PROM from previous findings conducted 4.1% in Mizan-Aman general hospital ( Endale et al., 2016) and 7.2% in Jimma (Bekabil et al., 2015) south west Ethiopia considering confidence interval (Za/2)= 95%=1.96 and level of precision =3% of the following formula.

$$n= \frac{Z\frac{\alpha^{2}}{2}p\left( 1-p \right)}{d^{2}}$$

The calculated sample size by using the above assumptions was 168 and 285 respectively. While after this, we are checked sample adequacy using double population formula from recent similar evidence conducted in Ethiopia as stated in the table below.

| Factor associated with PROM | Proportion unexposed group | Confidence interval (CI) | Power | Odds ratio | Ratio | Sample size |
| --- | --- | --- | --- | --- | --- | --- |
| Abortion history (Assefa et al., 2018) | 10.6% | 95% | 80% | 3.82 | 1 | 140 |
| Previous PROM (Assefa et al., 2018) | 6.2% | 95% | 80% | 7.64 | 1 | 80 |
| History caesarian section (Assefa et al., 2018) | 3.4% | 95% | 80% | 4.24 | 1 | 296 |
| Inter pregnancy interval (Workineh et al., 2018) | 63.23 | 95% | 80% | 0.31 | 1 | 110 |

Finally, after factor analysis of sample adequacy checked the larger sample size was taken from double population formula in the table. The final sample size for this study was 296.

**Thank you‼!**
