# Supplementary material for: Pre-labor rupture of membranes and associated factors among pregnant women admitted to the maternity ward, Northwest Ethiopia
Source: PLOS Glob Public Health. 2023 Mar 8;3(3):e0001702. doi: 10.1371/journal.pgph.0001702 (PMC10022274; doi:10.1371/journal.pgph.0001702)
Supplement: Attachment — Submitted filename: Response to Reviewers.docx [file pgph.0001702.s003.docx]

**Response to Reviewers**

We are happy for your constructive comments and suggestions. We are also learned a lot from these substantial scientific comments to improve the quality of this manuscript. We are addressed your comments and suggestions as stated below. The manuscript was revised as indicated in the highlights and track changes included in the main document.

**Comments to the Author**

1. If the authors have adequately addressed your comments raised in a previous round of review and you feel that this manuscript is now acceptable for publication, you may indicate that here to bypass the “Comments to the Author” section, enter your conflict of interest statement in the “Confidential to Editor” section, and submit your "Accept" recommendation.

Reviewer #1: (No Response)

Reviewer #2: (No Response)

**Thank you!**

2. Does this manuscript meet PLOS Global Public Health’s [publication criteria](https://journals.plos.org/globalpublichealth/s/criteria-for-publication)? Is the manuscript technically sound, and do the data support the conclusions? The manuscript must describe methodologically and ethically rigorous research with conclusions that are appropriately drawn based on the data presented.

Reviewer #1: No

Reviewer #2: Partly

**Thank you!** We are corrected on the entire document.

3. Has the statistical analysis been performed appropriately and rigorously?

Reviewer #1: No

Reviewer #2: Yes

**Thank you!** We are done the analysis using the data sets that has been supplied as supplementary files and we did it to the best of ours.

4. Have the authors made all data underlying the findings in their manuscript fully available (please refer to the Data Availability Statement at the start of the manuscript PDF file)?
The [PLOS Data policy](https://journals.plos.org/globalpublichealth/s/data-availability) requires authors to make all data underlying the findings described in their manuscript fully available without restriction, with rare exception. The data should be provided as part of the manuscript or its supporting information, or deposited to a public repository. For example, in addition to summary statistics, the data points behind means, medians and variance measures should be available. If there are restrictions on publicly sharing data—e.g. participant privacy or use of data from a third party—those must be specified.

Reviewer #1: Yes

Reviewer #2: Yes

**Thank you!**

5. Is the manuscript presented in an intelligible fashion and written in Standard English?
PLOS Global Public Health does not copyedit accepted manuscripts, so the language in submitted articles must be clear, correct, and unambiguous. Any typographical or grammatical errors should be corrected at revision, so please note any specific errors here.

Reviewer #1: No

Reviewer #2: No

**Thank you!** We are accepted your comment and revised the language of the entire document.

6. Review Comments to the Author

Please use the space provided to explain your answers to the questions above. You may also include additional comments for the author, including concerns about dual publication, research ethics, or publication ethics. (Please upload your review as an attachment if it exceeds 20,000 characters)

**Reviewer #1**

**Abstract:**

- Lines 44-45 The sentence should be rewritten: “Hence, we will reduce fetal and prenatal complications by giving attention to pregnant mothers with identified factors”, since the reduction in fetal and/or maternal complications also depend on a range of factors and intervention and not only by “giving attention …”

**Thank you!** We are accepted your scientific comment and corrected it accordingly in page 2 lines 44-46.

- Line 38: “The prevalence of PROM in this finding was 19%” should be “The prevalence of PROM in this study was 19%”

**Thank you!** We are accepted your comment and corrected as your suggestion page 2 line 38.

- Line 43: “pre-labor rapture of the membrane” should be “pre-labor rupture of the membranes”

**Thank you!** We are corrected as your comment page 2 line 43.

- Line 47: "Author summary?"

**Thank you!** We are corrected your comment accordingly page 3 line 48.

- Line 52: define the abbreviation of PPROM and make clear the difference between PROM and PPROM.

**Thank you!** We are accepted your comment and corrected in the document page 3 lines 51-52.

**Methods:**

- Line 119: “Finally, a total of 326 study participants ...": pls change to “… need to be recruited”?

**Thank you!** We are corrected as your suggestion and included in the document page 6 line 131.

- In the previous version of the manuscript, the editor commented “the formula of sample size calculation, ..., should be showed by the formula (not presenting in text) following the cross-sectional study design ... You could use p estimation from previous studies to calculate sample size for your study”. While being explained within the response to the editor and reviewers, that requested formula has not been incorporated into the revised version and needs to be inserted.

**Thank you!** The sample size for this study was computed through using double population formula Epi Info^TM^ version 7 from previous finding. Because of the primary objective using p-estimation yield insufficient sample size compared to sample adequacy checked using factor analysis of selected variables. This is the procedures we followed, we are trying to calculate sample size using single population proportion formula taking the proportion of pregnant mothers with PROM from previous findings conducted in Ethiopia 4.1% in Mizan-Aman general hospital ( Endale et al., 2016) and 7.2% in Jimma (Bekabil et al., 2015) considering the assumptions; 95% confidence interval (Za/2) =1.96 and level of precision =3% by using the following formulas.

$$n= \frac{Z\frac{\alpha^{2}}{2}p\left( 1-p \right)}{d^{2}}$$

The calculated sample size by using the above assumptions including 10% non-response rate was 185 and 314 respectively. We are also checked sample size adequacy by using factor analysis methods using previous findings statistically significant variables as stated in the table below.

| Factor associated with PROM | Proportion unexposed group | Confidence interval (CI) | Power | Odds ratio | Ratio | Sample size |
| --- | --- | --- | --- | --- | --- | --- |
| Abortion history (Assefa et al., 2018) | 10.6% | 95% | 80% | 3.82 | 1 | 140 |
| Previous PROM (Assefa et al., 2018) | 6.2% | 95% | 80% | 7.64 | 1 | 80 |
| History caesarian section (Assefa et al., 2018) | 3.4% | 95% | 80% | 4.24 | 1 | 296 |
| Inter pregnancy interval (Workineh et al., 2018) | 63.23 | 95% | 80% | 0.31 | 1 | 110 |

The sample size computed from single population was small, whereas the sample size using double population formula was large which is considered as adequate sample. The final sample size for this study by considering 10% non-response rate computed from double population formula was 326.

- Lines 126-127: Are the cases having rupture of membranes confirmed only by interviews conducted by midwives and not by vaginal examination? Is there any test of confirmation for the PROM being implemented? Please elaborate this most important modality and examination for confirmation of PROM in clinical setting.

**Thank you!** In this study outcome variable PROM have been evaluated using maternal chart review which is already documented after necessary diagnosis tests done in the hospital. Please remember, in this study we did not done any investigation parameters. Despite, we are included in the document diagnosis modalities and tests available in the hospital page 6 lines 119-121.

**Results & Discussion:**

- Table 2: The authors presented data on polyhydramnios condition. How was the polyhydramnios assessed and/or confirmed? By ultrasound examination or by obstetrical examination? Quantitative or semi-quantitative modality?

**Thank you!** We are used already documented maternal charts of such clinical variables. Please, consider that in this study we did not done any investigation parameters to assess variables. Even though, we are included in the document available diagnosis procedures will be done for pregnant women in the hospital page 6 lines 119-121.

- Fetal position could be an influencing factor, abnormal fetal position near term could be a contributing factor to PROM status. Please provide more discussion.

**Thank you!** We addressed your concern but it is not statistical significant. We are included possible explanations in the document page 16 lines 265-268.

**Reviewer #2**

1. **Introduction**

- The introduction part has come from global to local going through the prevalence of the PROM. But there are language (grammatic) gaps that needs to be edited so that the ultimate message of the session will be clear for the readers. (eg. Line 76 …”multiparty mothers” need be multi para mothers, line 79 ………this is also 14.6%....had better re phrased to there is also research done in ……. Line 85 the punctuation (and 11% in term deliveries with this, 40-75% were neonatal deaths [9, 11]) and grammatic fluency need to be re phrased. Line 89…. “increase 10 fold in neonatal death” better stated in such a way that make sense….….PROM increase neonatal death by 10 folds…….

**Thank you for your constructive comments!** We are accepted your comment and corrected accordingly as your suggestions page 4 lines 72-79 and 89.

- Line 246…… individual chart this might be introduced information bias,” I think wanted to mention individual chart which will introduce information bias. There are many researches being conducted on secondary data with ethical clearance and still we can’t complain information bias by reviewing the chart.

**Thank you!** We are accepted and included in the document page 16 lines 270-273.

The session also has gone through the associated factors of the PROM. It has also put the rationale for this study mentioning there are no adequate investigation of associated factors for PROM that the study aimed to address it.

**Areas to be improved are:**

I. There are many studies done on PROM in Ethiopia, few from many are: Getahun Tiruye et al. prevalence of PROM and it associated factor in Ethiopia, Dagne Addisu et al. Prevalence of PROM and its’ associated factors among admitted pregnant women in Debre Tabor General Hospital, Belayneh Hamdela et al. incidence of PROM and its association with pregnancy interval…etc are done in Ethiopia. So, for this study I don’t think that there is inadequacy of the evidence. So, the authors had better re statement this portion.

**Thank you!** We are accepted your comment and included the possible justification in the document page 5 lines 99-104.

II. All of the factors mentioned in the session are expected to be investigated and discussed in the result part of this study. Factors that are not discussed in the discussion part of this study need to be removed from this session.

**Thank you!** We are corrected as your recommendation and included in page 5 lines 96-99.

III. The statements from line 82-93 reached mainly the complication of PROM specifically on neonates after delivery. Since the aim of this study is reaching only the prevalence of PROM and its’ associated factor, there is no reason that the Authors need to go through the complication of the PROM.

**Thank you!** Sorry to say, we are regret to accept this comment. Because we are stated the complication of PROM in this document is mainly to depict the extent of severity of PROM resulted neonatal complications.

**Method and material part:**

Again the study has gone through each steps well detailed. Study area was well described that the study hospital is referral hospital where selected cases are referred to from health facilities. Source population was mentioned well and exclusion criteria was mentioned too. Sampling method is fine and assumptions were well mentioned.

**Area where need to be revised:**

1. It would have been very well if the authors also mention what were their study population and mention clearly what kind of the pregnant women are the source population mainly contains.

**Thank you!** For this study, pregnant women admitted in the maternity ward were considered as source population and included in the document page 6 lines 117-119 and 121-123.

2. I feel that the cases attending ANC, maternity admission in the hospitals are high risk mothers which affect the result of the study and sampling methods as only high risk are being considered as source population.

**Thank you!** We are accepted your comment and included your comment in the limitation of the study in the page 16 lines 270-273.

3. Line 121 to 122 …..”By considering the hospital recent three months from January to  march 2019 data” is not clear statement. Does it mean the hospital admission or hospital attendance, delivery or something different has to be mentioned clearly.

**Thank you!** We are corrected and included in the document page 6 lines132-135.

4. As the sampling method considered two population (case and none case) …..so are the authors going to tell us incidence among cases and none cases, compare the two group? In general it would be great if the authors would tell us the rationale behind using the two population formula.

**Thank you!** The sample size for this study was computed through using double population formula Epi Info^TM^ version 7 from previous finding. Because of the primary objective using p-estimation yield insufficient sample size compared to sample adequacy checked using factor analysis of selected variables. This is the procedures we followed, we are trying to calculate sample size using single population proportion formula taking the proportion of pregnant mothers with PROM from previous findings conducted in Ethiopia 4.1% in Mizan-Aman general hospital ( Endale et al., 2016) and 7.2% in Jimma (Bekabil et al., 2015) considering the assumptions; 95% confidence interval (Za/2) =1.96 and level of precision =3% by using the following formulas.

$$n= \frac{Z\frac{\alpha^{2}}{2}p\left( 1-p \right)}{d^{2}}$$

The calculated sample size by using the above assumptions including 10% non-response rate was 185 and 314 respectively. We are also checked sample size adequacy by using factor analysis methods using previous findings statistically significant variables as stated in the table below.

| Factor associated with PROM | Proportion unexposed group | Confidence interval (CI) | Power | Odds ratio | Ratio | Sample size |
| --- | --- | --- | --- | --- | --- | --- |
| Abortion history (Assefa et al., 2018) | 10.6% | 95% | 80% | 3.82 | 1 | 140 |
| Previous PROM (Assefa et al., 2018) | 6.2% | 95% | 80% | 7.64 | 1 | 80 |
| History caesarian section (Assefa et al., 2018) | 3.4% | 95% | 80% | 4.24 | 1 | 296 |
| Inter pregnancy interval (Workineh et al., 2018) | 63.23 | 95% | 80% | 0.31 | 1 | 110 |

The sample size computed from single population was small, whereas the sample size using double population formula was large which is considered as adequate sample. The final sample size for this study by considering 10% non-response rate computed from double population formula was 326.

**Result part:**

- The associated factors variables that are mentioned rigorously and cross tabulated well. Areas where need to be improved:

1. Most of the associated factors mentioned in the literature are not addressed here, eg. Coffee drinking, smoking, maternal weight gain, birth interval are not mentioned assessed here too.

**Thank you!** We are accepted your scientific comment but our tool dose not accompanied these variables and sorry, we did not addressed your concerns in this study.

2. Factors that are mentioned, investigated and included in the result part are not included in the literature review, introduction part which make the authors to come to the conclusion that these factors are not adequately assessed in the country.

**Thank you!** We are accepted your comment and corrected in the document page 5 lines 96-99.

3. Since authors interviewed the clients how did the authors able to address the fetal alignment is not clear, It would be great if it is clearly mentioned weather ultrasounds were used to know and at what gestational age it was taken. Because oblique and transverse presentation in preterm (28-34wks) is quite common and usually it is normal to have none cephalic presentation for 1^st^ and second trimester pregnancy.

**Thank you!** In this study we used maternal chart review those variables needs an investigation of parameters. We are accepted your comment and included the available different diagnosis modalities in the hospital page 6 lines 119-121.

**Discussion part:**

1. The prevalence of specific health problem can be gained from the general population not from the specially collected population. The source population are all high risk mothers as they are referred for special care at the hospital. So, this study is mainly talking about prevalence among mothers attending Debre Markos hospital.

**Thank you!** We are accepted your comment and included your concern as the limitation of the study in the document page 16 lines 270-273.

2. Line 198-199, the difference between the prevalence of this study and others studies mentioned claims “previous study findings were extracted either of face to face interviews or individual chart reviews …..“ is not clear as this study were also conducted face to face interview as mentioned in the data collection method. So, the authors had better re statement and reason out in better way why different.

**Thank you!** We are accepted your comment and included in the document page 14 lines 208-221.

3. Line 215 -218, reasoning for high PROM rate in preterm mentioned malpresentation but in this investigation oblique and transvers presentation were not found to be factor for PROM. Again, the authors had better see into it and re investigate the reason behind.

**Thank you!** We are checked but it is not statistically significant against previous evidence. We are mentioned in the discussion variables that are significant in the previous evidence but not in this study we are included in the page 16 lines 265-268.

4. Overall the finding of this study is similar with studies conducted all over the world including researches done in many parts of Ethiopia. For the factors related to PROM are same with the book factors and already known and investigated once what the authors think is the necessity of this study had better well detailed and explained in the introduction part. So, the significance of this study will be more clear.

**Thank you!** We are included the finding significance in the document page 5 lines 104-107.

**Conclusion part:**

1. The prevalence in this study is point prevalence in Hospital case attending or admitted patients and not in general population which makes it not in a position to compare it with the world prevalence of PROM which is in general population (pregnant mothers).

**Thank you!** We are included your concern in the limitation section of the study and conclusion in the page 16-17 lines 270-275.

2. Line 251-253 the recommendation part is not on the factors that the study addressed. This study didn’t assess the attention status of the health care workers and other responsible bodies and poor focus from health workers and responsible bodies were not finding of this study. So, Authors had better recommend based on their study finding.

**Thank you!** We are accepted your comment and corrected in the document page 17 lines 278-282.

7. PLOS authors have the option to publish the peer review history of their article ([what does this mean?](https://journals.plos.org/globalpublichealth/s/editorial-and-peer-review-process#loc-peer-review-history)). If published, this will include your full peer review and any attached files.

**Do you want your identity to be public for this peer review?** If you choose “no”, your identity will remain anonymous but your review may still be made public.

For information about this choice, including consent withdrawal, please see our [Privacy Policy](https://www.plos.org/privacy-policy).

Reviewer #1: No

Reviewer #2: **Yes:** Abela, Abebe Negesso (BSc,MD,MPH)

**Thank you for your constructive comments and suggestions‼!**
