# Supplementary material for: Pre-labor rupture of membranes and associated factors among pregnant women admitted to the maternity ward, Northwest Ethiopia
Source: PLOS Glob Public Health. 2023 Mar 8;3(3):e0001702. doi: 10.1371/journal.pgph.0001702 (PMC10022274; doi:10.1371/journal.pgph.0001702)
Supplement: Attachment — Submitted filename: Response to Reviewers.docx [file pgph.0001702.s004.docx]

**Response to Reviewers**

We are glad for your constructive comments and suggestions. We are addressed your comments and suggestions as stated below by point-by-point response.

**Journal Requirements:**

1. Please review your reference list to ensure that it is complete and correct. If you have cited papers that have been retracted, please include the rationale for doing so in the manuscript text or remove these references and replace them with relevant current references. Any changes to the reference list should be mentioned in the rebuttal letter that accompanies your revised manuscript. If you need to cite a retracted article, indicate the article’s retracted status in the References list and also include a citation and full reference for the retraction notice.

**Thank you!** We are checked all references only **“**Dutta D: **DC Dutta’s: Textbook of Obstetric including perinatology and contraception. Vol 46**: The Health Sciences Publisher; 2015”is retracted and removed this in the lists. Instead of this retracted, we are included new reference “Konar H: **DC Dutta's textbook of obstetrics**, 8^th^ edition. New Delhi: Jaypee Brothers Medical Publishers; 2018.” There is also unpublised hospital report included in the reference list inorder to support the study setting information.

2. We have noticed that you have uploaded Supporting Information files, but you have not included a list of legends. Please add a full list of legends for your Supporting Information files after the references list.

**Thank you!** We are included your comment in the clean document page 25.

Additional Editor Comments (if provided):

1. Check Table 2 to make sure your sample size of study is 315 for data analysis, also check this consistent sample size in overall manuscript (not 326 in total number).

**Thank you!** In this document there is no sample size consistency problem. The calculated sample size was 326 but only 315 study participants with 97% of response rate were participated in the study. This statement was included in result section clean document page 7 line 159.

2. Add evidence of ethical approval document in part of Ethical considerations, specify Number and the date of document signed by IRB.

**Thank you!** We are included your comment in the clean document page 24 lines 449-450.

**Comments to the Author**

1. If the authors have adequately addressed your comments raised in a previous round of review and you feel that this manuscript is now acceptable for publication, you may indicate that here to bypass the “Comments to the Author” section, enter your conflict of interest statement in the “Confidential to Editor” section, and submit your "Accept" recommendation.

Reviewer #1: (No Response)

Reviewer #2: (No Response)

**Thank you!**

2. Does this manuscript meet PLOS Global Public Health’s [publication criteria](https://journals.plos.org/globalpublichealth/s/criteria-for-publication)? Is the manuscript technically sound, and do the data support the conclusions? The manuscript must describe methodologically and ethically rigorous research with conclusions that are appropriately drawn based on the data presented.

Reviewer #1: Yes

Reviewer #2: Partly

**Thank you!** As of our best of knowledge we are addressed the reviewers’ comments and suggestions in the document to meet the publication criteria till these three revisions.

3. Has the statistical analysis been performed appropriately and rigorously?

Reviewer #1: Yes

Reviewer #2: No

**Thank you!** We are supplied the data sets as supplemental information to ensure the trustworthiness of the statistical analysis. We did the statistically rigorously and reproduceable.

4. Have the authors made all data underlying the findings in their manuscript fully available (please refer to the Data Availability Statement at the start of the manuscript PDF file)?
   The [PLOS Data policy](https://journals.plos.org/globalpublichealth/s/data-availability) requires authors to make all data underlying the findings described in their manuscript fully available without restriction, with rare exception. The data should be provided as part of the manuscript or its supporting information, or deposited to a public repository. For example, in addition to summary statistics, the data points behind means, medians and variance measures should be available. If there are restrictions on publicly sharing data—e.g. participant privacy or use of data from a third party—those must be specified.

Reviewer #1: Yes

Reviewer #2: Yes

**Thank you!**

5. Is the manuscript presented in an intelligible fashion and written in standard English?

   PLOS Global Public Health does not copyedit accepted manuscripts, so the language in submitted articles must be clear, correct, and unambiguous. Any typographical or grammatical errors should be corrected at revision, so please note any specific errors here.

Reviewer #1: No

Reviewer #2: No

**Thank you!** We are revised extensively the English language to the entire document.

6. Review Comments to the Author
   Please use the space provided to explain your answers to the questions above. You may also include additional comments for the author, including concerns about dual publication, research ethics, or publication ethics. (Please upload your review as an attachment if it exceeds 20,000 characters)

**Thank you!**

Reviewer #1: The authors have made substantial revisions within the R2 version.
There are still some points of concern that have been raised or newly emerged that need to be solved by the authors:

- Lines 126-135: please elaborate and made a consistent revision to the sample size calculation and the sample size estimate formula (to be added to the manuscript) as the authors mentioned in an appropriate within the Response to the Reviewers section.

**Thank you!** As we clearly described in the previous responses to the reviewers, the authors estimated the required sample size using single population proportion and double population formula using previous studies conducted in Ethiopia. The larger sample size is considered as the final sample size which is estimated using double population formula as clearly described in the document. Sorry to say that, after detail discussion of the authors decided that we did not accept your recommendation to include in the document. Instead, we are included the statement that describe the attempts of how we calculate the sample size in the clean document page 6 lines 123-125.

Lines 142-144: “PROM is confirmed clinically (painless gush of fluid leaking from the vagina and a change in color or a decrease in the size of the uterus”: the change in color of what? of the fluid? a decrease in the size of the uterus is not the clinical sign of PROM, unless it’s a severe polyhydramnios case.

**Thank you!** We are accepted your comment and revised the diagnosis modalities of PROM in the clean document pages 6-7 and lines 139-141.

- Lines 216-217: “and India 7.69 of primiparity and 10.41 of multipara”: check syntax and “%” character.

**Thank you!** We are accepted the comment and corrected as your recommendation.

- Lines 278-279: “Strengthen counseling, early testing, and treatment including partners for sexually transmitted 279 infections before conception”: check syntax for this sentence.

**Thank you!** We are accepted the comment and revised in the clean document page 16 lines 273-275.

Reviewer #2: The language part i recommend the manuscript edited by someone whose English language is native or another known editor.

**Thank you!** We are revised extensively the English language to the entire document.

I hope the authors address those major comments i have mentioned on the attached document.

7. PLOS authors have the option to publish the peer review history of their article ([what does this mean?](https://journals.plos.org/globalpublichealth/s/editorial-and-peer-review-process#loc-peer-review-history)). If published, this will include your full peer review and any attached files.

   **Do you want your identity to be public for this peer review?** If you choose “no”, your identity will remain anonymous but your review may still be made public.
   For information about this choice, including consent withdrawal, please see our [Privacy Policy](https://www.plos.org/privacy-policy).

Reviewer #1: No

Reviewer #2: No

**Thank you!**

Reviewer #2

Abstract:

- line 28, evidence in Amhara is limited can’t be a justification as there are adequate information online, better to put other realistic justification.

**Thank you!** We are accepted your comment and included in the clean document page 2 lines 28-29.

- Line 29: prevalence of a disease can’t be determined from institutional based study.

**Thank you!** We are accepted your comment and changed the term prevalence to magnitude to the entire document.

- Line 38-42 (the result part of the body) findings are entirely not different form other studies conducted in Ethiopia, or literatures used in this study. Therefore, it is expected for the authors to justify why this study was important to conduct, what is the different finding, why the publication of this paper is necessary for no different finding.

**Thank you!** We are clearly described the justification and significance of the study in the clean document page 5 lines 97-106.

- Line 43-46 I have not seen any Sexually transmitted infection in the result part. What I saw is abnormal vaginal discharge which in most case be bacterial vaginosis which is not STI. So, where STI is coming in the conclusion is not clear making it difficult to understand the conclusion part.

**Thank you!** We are corrected this and included in the clean document.

- Line 107 is telling that the aim of the study is to determine the prevalence of the PROM, line 121-123 the source population are the admitted pregnant mothers. This two can’t go together this is just share of PROM among admission and can’t tell even the prevalence of prom in the hospital. To study the prevalence of PROM in the hospital source population has to be all pregnant mothers in hospital weather admitted or not (all ANC following mothers), If the study aims to measure prevalence in the region, zone or woreda the source population has to be all pregnant mothers in the region weather having ANC, admitted or have no ANC at all (community pregnant mothers).

**Thank you!** We are changed the term prevalence to magnitude in the entire document. In this study the source population was all pregnant women admitted to the maternity ward for delivery or contemporary management services. Since the health facility is referral hospital, we are tried to determine the magnitude among those risk population segments. This may overestimate the magnitude, but we are considered this in the limitation of the study.

**Thank you for your constructive comments and suggestions‼!**
