# Supplementary material for: Pre-labor rupture of membranes and associated factors among pregnant women admitted to the maternity ward, Northwest Ethiopia
Source: PLOS Glob Public Health. 2023 Mar 8;3(3):e0001702. doi: 10.1371/journal.pgph.0001702 (PMC10022274; doi:10.1371/journal.pgph.0001702)
Supplement: Attachment — Submitted filename: Response to Reviewers.docx [file pgph.0001702.s005.docx]

**Response to Reviewers**

Thank you for your constructive comments and suggestions. We are addressed your comments and suggestions as stated below by point-by-point response.

Journal Requirements:

- Please review your reference list to ensure that it is complete and correct. If you have cited papers that have been retracted, please include the rationale for doing so in the manuscript text,or remove these references and replace them with relevant current references. Any changes to the reference list should be mentioned in the rebuttal letter that accompanies your revised manuscript. If you need to cite a retracted article, indicate the article’s retracted status in the References list and also include a citation and full reference for the retraction notice.

**Thank you!** We have checked all the reference and there is no retracted reference lists in this mnuscript. All references are available in google scholar and other databases but this three references lists are avaialable on google search engine with links.

Sultana S, Ishtiaq S, Malik U, Akhai AZ, Nadeem K: **Maternal and perinatal outcome in**

**preterm pre-labor rupture of membranes**. *Pakistan Journal of Surgery* 2019, **35**(1):73-77.

<http://www.pjs.com.pk/journal_pdfs/jan_mar19/73.pdf>

Woyessa T, Fulea L, Edossa A: **Premature rupture of the membrane and its associated factors among pregnant women admitted to public Hospitals in Nekemte town, Western Ethiopia**. *International Research Journal of Obstetrics and Gynecology* 2020, **3**:27. <https://escipub.com/irjog-2019-12-2905/>

Addisu D, Melkie A, Biru S: **Prevalence of preterm premature rupture of membrane and its**

**associated factors among pregnant women admitted in Debre Tabor General Hospital,**

**North West Ethiopia: Institutional-based cross-sectional study**. *Obstetrics and Gynecology*

*International* **2020**.

<https://scholar.google.com/scholar?hl=en&as_sdt=0%2C5&q=+Prevalence+of+preterm+premature+rupture+of+membrane+and+its+associated+factors+among+pregnant+women+admitted+in+Debre+Tabor+General+Hospital%2C+North+West+Ethiopia%3A+Institutional-based+cross-sectional+study.+Obstetrics+and+Gynecol&btnG=>
**Comments to the Author**

1. If the authors have adequately addressed your comments raised in a previous round of review and you feel that this manuscript is now acceptable for publication, you may indicate that here to bypass the “Comments to the Author” section, enter your conflict of interest statement in the “Confidential to Editor” section, and submit your "Accept" recommendation.

Reviewer #2: All comments have been addressed.

Reviewer #3: All comments have been addressed.

**Thank you!**

2. Does this manuscript meet PLOS Global Public Health’s [publication criteria](https://journals.plos.org/globalpublichealth/s/criteria-for-publication)? Is the manuscript technically sound, and do the data support the conclusions? The manuscript must describe methodologically and ethically rigorous research with conclusions that are appropriately drawn based on the data presented.

Reviewer #2: Yes

Reviewer #3: Yes

**Thank you!**

3. Has the statistical analysis been performed appropriately and rigorously?

Reviewer #2: Yes

Reviewer #3: Yes

**Thank you!**

4. Have the authors made all data underlying the findings in their manuscript fully available (please refer to the Data Availability Statement at the start of the manuscript PDF file)?

   The [PLOS Data policy](https://journals.plos.org/globalpublichealth/s/data-availability) requires authors to make all data underlying the findings described in their manuscript fully available without restriction, with rare exception. The data should be provided as part of the manuscript or its supporting information, or deposited to a public repository. For example, in addition to summary statistics, the data points behind means, medians and variance measures should be available. If there are restrictions on publicly sharing data—e.g. participant privacy or use of data from a third party—those must be specified.

Reviewer #2: Yes

Reviewer #3: Yes

**Thank you!**

5. Is the manuscript presented in an intelligible fashion and written in standard English?

   PLOS Global Public Health does not copyedit accepted manuscripts, so the language in submitted articles must be clear, correct, and unambiguous. Any typographical or grammatical errors should be corrected at revision, so please note any specific errors here.

Reviewer #2: Yes

Reviewer #3: Yes

**Thank you!**

6. Review Comments to the Author

Please use the space provided to explain your answers to the questions above. You may also include additional comments for the author, including concerns about dual publication, research ethics, or publication ethics. (Please upload your review as an attachment if it exceeds 20,000 characters)

Reviewer #2: I can't comment better for the language, and you may have invited someone native of English.

**Thank you!**

Reviewer #3: Abstract

- Introduction - Unclear sentence “Despite a paucity of evidence on the magnitude and the factors affecting PROM after 28 weeks of gestation to before the onset of labor”.

**Thank you!** We are corrected the comment and included on the clean document page 2 lines 29-30.

**Note:** This study included both preterm premature rupture of membranes (PPROM) occurs before 37 weeks of gestation (this means completed 28 weeks to 37 weeks) and term PROM after 37 weeks of gestation but before onset of labor.

Methods

- Sample size determination shall be written separately.

**Thank you!** We are corrected as your comment on the clean document page 6.

- The sampling procedure shall be explained a bit more mentioning patient flow.

**Thank you!** We are corrected as your comment on the clean document page 6-7 lines 134-136 in addition we are also clearly described this information on page 6 lines 116-120

- How PROM status is asserted in this study is not mentioned. Whether by physical examination or from chart review.

**Thank you!** In this study data was collected by face-to-face interview for socio-demographic variables and chart review for important clinical variables including outcome ascertainment. PROM status was evaluated using individual chart review as described on the clean document page 7 lines 140-145.

Results

- The magnitude of the problem under study is mentioned in Socio-demographic and economic characteristics section and needs to be written separately.

**Thank you!** We are corrected as your recommendation and included on clean document page 12 lines 188-193.

- History of medical illness and clinical related factors and Obstetrics and gynecological-related factors shall be presented as characteristics do not factor.

**Thank you!** We are corrected as your recommendation on the clean document.

- The * in the foot note of table 4 is not important as the numbers are mentioned in the column titled p-value.

**Thank you for your concern!** As mentioned in the table, there are variables with p-value <0.05. Polyhydramnios and history of abnormal vaginal discharge were reported the p-value 0.000. So that, we are using * in the foot note to indicate the p-value 0.000 reported as <0.001.

Discussion

- It has been mentioned in the first paragraph as the high magnitude of PROM in this study with other studies in Addis Ababa and Harar are due to the type of setting as “This similarity might be due to the study settings, which were conducted in referral hospitals, which increase the high risk of pregnant women being referred from different health facilities for better diagnosis and management.” But also it is mentioned that this finding is much higher than studies in “Tikur Ambesa teaching hospital 1.4% [24], Jimma 7.2% [34], Mizan Aman Hospital 4.1% [22], Nekemte town public hospitals 13.4% [21]” which are also referral hospitals receiving high risk mothers. This makes your justification to the finding questionable.

**Thank you!** We are accepted the comment and corrected on the clean document page 14 lines 214-215.

- The study’s finding that those mother with a gestational age 37-40 weeks are less likely to have PROM than those less than 37 weeks of gestation is explained by the statement “When the body prepares for normal labor and delivery, fetal membranes become weakened and fragile.” Do you think this is logical?

**Thank you for your critical citation!** We are accepted the comment and removed this information in the document.

**Thank you again for your constructive comments!!!**
